# Role of Endoplasmic Reticulum Stress-Autophagy Axis in Severe Burn-Induced Intestinal Tight Junction Barrier Dysfunction in Mice

**DOI:** 10.3389/fphys.2019.00606

**Published:** 2019-05-22

**Authors:** Yalan Huang, Yu Wang, Yanhai Feng, Pei Wang, Xiaochong He, Hui Ren, Fengjun Wang

**Affiliations:** ^1^ School of Nursing, Third Military Medical University (Army Medical University), Chongqing, China; ^2^ State Key Laboratory of Trauma, Burns and Combined Injury, Institute of Burn Research, Southwest Hospital, Third Military Medical University (Army Medical University), Chongqing, China; ^3^ Department of Gastroenterology, Southwest Hospital, Third Military Medical University (Army Medical University), Chongqing, China

**Keywords:** burn injury, endoplasmic reticulum stress, autophagy, tight junction, intestinal barrier dysfunction

## Abstract

Severe burn injury induces intestinal barrier dysfunction; however, the underlying mechanisms remain elusive. Our previous studies have shown that the intestinal epithelial tight junction (TJ) barrier dysfunction is associated with both endoplasmic reticulum (ER) stress and autophagy in severely burned mice, but the precise role of ER stress and autophagy in the burn-induced intestinal TJ barrier dysfunction needs to be determined. In this study, female C57/BL6 mice were assigned randomly to either sham burn or 30% total body surface area (TBSA) full-thickness burn. The effects of ER stress and autophagy on the intestinal epithelial TJ barrier were validated by inducing or inhibiting both ER stress and autophagy in mice treated with sham burn or burn injury. The intestinal permeability, expression, and localization of TJ proteins, ER stress, and autophagy were assessed by physiological, morphological, and biochemical analyses. The results showed that inducing ER stress with tunicamycin or thapsigargin caused the activation of autophagy, the increase of intestinal permeability, as well as the reduction and reorganization of TJ proteins in the sham-burned mice, and aggravated the burn-induced activation of autophagy, increase of intestinal permeability, as well as the reduction and reorganization of TJ proteins. In contrast, inhibiting ER stress with 4-phenylbutyrate alleviated the burn-induced activation of autophagy, increase of intestinal permeability, as well as the reduction and reorganization of TJ proteins. In addition, inducing autophagy with rapamycin resulted in the increase of intestinal permeability, as well as the reduction and reorganization of TJ proteins in the sham-burned mice, and aggravated the burn-induced increase of intestinal permeability as well as the reduction and reorganization of TJ proteins. However, inhibiting autophagy with 3-methyladenine attenuated the burn-induced increase of intestinal permeability, as well as the reduction and reorganization TJ proteins. It is suggested that the ER stress-autophagy axis contributes to the intestinal epithelial TJ barrier dysfunction after severe burn injury.

## Introduction

The intestinal epithelia form an important barrier between the intestinal mucosa and the luminal environment. The intestinal epithelial barrier, when intact, functions to resist the invasion of intraluminal bacteria, endotoxins, and other pathogens. The apical intercellular tight junctions (TJs) are essential for the intact paracellular barrier function. It is well known that increased permeability caused by the disruption of the intestinal epithelial TJ barrier is crucial for the development of intestinal inflammation (Arnott et al., 2000; Arrieta et al., 2009; Turner, 2009). We, along with other investigators, have found that severe burn injury directly causes the loss of intestinal barrier function and then leads to increased intestinal permeability (Deitch, 1990; Earley et al., 2015), both of which contribute to the development of systemic inflammation and multiple organ failure in severe burn injury (Magnotti and Deitch, 2005; Arrieta et al., 2009; Chen et al., 2012; Clark et al., 2017). Although the importance of burn-induced intestinal barrier disruption is recognized, the mechanisms of intestinal barrier dysfunction after severe burn injury remain poorly understood.

Endoplasmic reticulum (ER), as a membrane-bound organelle, plays a crucial role in folding of secreted and membrane proteins. ER stress triggers the unfolded protein response (UPR) to restore ER homeostasis toward reestablishing normal ER function through the mediation of three ER transmembrane sensors: activating the pancreatic ER stress kinase (PERK), inositol-requiring enzyme 1 (IRE1), and transcription factor 6 (ATF6) (Hosoi and Ozawa, 2009). Studies showed that excessive ER stress and impaired UPR signaling may cause inflammatory bowel diseases mainly through induction of intestinal epithelial cell apoptosis and intestinal proinflammatory response as well (Hiramatsu et al., 2015; Hosomi et al., 2015; Luo and Cao, 2015). Although we have found that the intestinal ER stress is significantly induced after severe burn injury (Huang et al., 2018), the contribution of ER stress to intestinal TJ barrier dysfunction is still not conclusive.

Autophagy is a catabolic process that degrades and recycles damaged organelles and misfolded proteins through the lysosomal machinery (Wirawan et al., 2012; Hale et al., 2013). Autophagy is active in the normal intestinal mucosa (Groulx et al., 2012); however, excessive autophagy and impaired autophagy participate in intestinal inflammation (Cooney et al., 2010; Wildenberg et al., 2012). We, along with other investigators, have shown that severe burn injury induced autophagy in some visceral organs such as the intestine (Huang et al., 2018), heart (Xiao et al., 2012), liver (Song et al., 2014), and lungs (Dong et al., 2017). The activation of autophagy has recently been reported to be associated with the epithelial barrier disruption in Caco-2 cell monolayers challenged with lipopolysaccharide (Feng et al., 2018). Accumulating data have revealed the bidirectional linkage between autophagy and ER stress (Yorimitsu et al., 2006; Hoyer-Hansen and Jaattela, 2007; Qin et al., 2014), and ER stress activates autophagy *via* the inhibition of mTOR, the induction of Atg12 expression, or the activation of JNK (Ogata et al., 2006; Yorimitsu et al., 2006; Kouroku et al., 2007). Although our previous study has revealed that intestinal barrier dysfunction is accompanied by the activation of both ER stress and autophagy following severe burn injury (Huang et al., 2018), the exact role of ER stress and autophagy in the regulation of severe burn-induced intestinal barrier dysfunction remains unknown.

In this *in vivo* study, we demonstrate that severe burn induces ER stress and then activates autophagy in intestinal epithelial cells of mice. The ER stress-induced activation of autophagy results in the intestinal TJ barrier dysfunction following severe burn injury.

## Materials and Methods

### Ethics Statement

The animal studies were approved by the Animal Care and Use Committee of the Third Military Medical University (Army Medical University), Chongqing, China. And all animal procedures were performed in adherence to protocols approved by the Ethics Committee of Southwest Hospital, Third Military Medical University (Army Medical University).

## Animal Model and Procedures

Healthy female C57BL6 mice (8–10 weeks old, weighing 20–25 g) were purchased from the Animal Center, Third Military Medical University (Army Medical University), fed with a standard rodent chow diet and watered and housed with a 12 h light/dark cycle. All the animals were allowed to acclimate for 1 week prior to the experiment and randomly divided into 12 groups: control (sham burn), tunicamycin (Tm), thapsigargin (Tg), 4-phenylbutyrate (4-PBA), rapamycin (RAPA), 3-methyladenine (3-MA), burn, burn+Tm, burn+Tg, burn+4-PBA, burn+RAPA, and burn+3-MA. The mice in the indicated groups were intraperitoneally injected with tunicamycin (1.0 mg/kg; Calbiochem, MA, USA; D00176186) (Dong et al., 2015), thapsigargin (300 ng/kg; Sigma, St Louis, MO; 112M4011V) (Zhang et al., 2014), 4-PBA (80 mg/kg; Calbiochem, MA, USA; 2626840) (Ayala et al., 2012; Kim et al., 2013), rapamycin (4 mg/kg; Sigma, St Louis, MO; 2853724) (Wagner et al., 2012; Zhang et al., 2017a,b), or 3-methyladenine (15 mg/kg; Sigma, St Louis, MO; 026M4190V) (Zhang et al., 2014), respectively, 1 h before burn or sham burn. Meanwhile, the mice in the control or burn group just received the same volume of saline. A well-established method was used to induce a 30% full-thickness scald burn (Huang et al., 2018). Mice were anesthetized with 1 g/L pentobarbital sodium (30 mg/kg body weight i.p.), and the dorsal and lateral surfaces were shaved. The dorsum was immersed in 90°C water for 10 s, and then lactated Ringer’s solution (1 ml) was immediately administered intraperitoneally for resuscitation. The sham-burned mice received the same procedures except that the temperature of water was 37°C. Finally, mice were housed in separate cages and anesthetized to monitor intestinal permeability at 6 h postburn. After that, the mice were immediately euthanized to collect the samples of ileum for histological, immunofluorescent, and immunoblot analysis, respectively.

### Monitoring Intestinal Paracellular Permeability

The intestinal paracellular permeability was determined as we described previously (Chen et al., 2012). Previous studies showed that intestinal permeability was sensitive in mouse ileum (Mazzon et al., 2002; Chen et al., 2012). Briefly, a laparotomy was performed under anesthesia, and a 5-cm segment of the ileum adjacent to the cecum was dissociated, with well-protected superior mesenteric vessels. A 2–0 silk suture tied the isolated ileum at its bilateral end; 0.2 ml of 0.1 mol/L phosphate-buffered saline (PBS, pH 7.2) containing fluorescein isothiocyanate-labeled dextran (FITC-dextran, 20 mg/ml) (Sigma, St. Louis, MO) was injected into the lumen. The blood sample was taken after 30 min and centrifuged at 4°C, 3,000 × *g*, for 10 min. The plasma was taken and diluted at 1:10 with PBS to measure the fluorescence intensity with a microplate reader (Varioskan Flash, Thermo Electron Corporation, Vantaa, Finland) with an excitation wavelength of 480 nm and an emission wavelength of 520 nm. The concentrations of plasma FITC-dextran were calculated from a standard curve generated by serial dilution of FITC-dextran in PBS.

### Immunoblot Analysis

Ileal mucosa scrapings were collected and homogenized in RIPA buffer. The lysate was centrifuged at 4°C, 15,000 × *g*, for 15 min, and the supernatants were collected for protein expression assessment. Protein concentration was determined using the *RC DC* protein assay kit (Bio-Rad, Hercules, CA). RC DC protein assay is a colorimetric assay for protein quantitation with all the functionality of the original DC protein assay. This assay is based on the Lowry assay but has been modified to be reducing-agent compatible as well as detergent compatible. Western blotting was performed as we reported (Huang et al., 2018); 30 mg protein was loaded for each lane and then separated on SDS-PAGE gel (6, 8, 10, or 12%) and transferred to a PVDF membrane (Millipore). Membranes were blocked using 5% non-fat milk at room temperature for 1 h and then probed with the following primary antibodies at 4°C overnight: ZO-1 (1:1,000; Invitrogen, cat. no. 33-9100, CA, USA), occludin (1:1,000; Invitrogen, cat. no. 71-1500, CA, USA), claudin-1 (1:1,000; Invitrogen, cat. no. 37-4490, CA, USA), Bip (1:1,000; Cell Signaling, cat. no. 3177, MA, USA), CHOP (1:1,000; Cell Signaling, cat. no. 2895, MA, USA), XBP1 (1:1,000; abcam, cat. no. ab37152, MA, USA), LC3B (1:1,000; Sigma-Aldrich, cat. no. L7543, MO, USA), p62 (1:1,000; Sigma-Aldrich, cat. no. P0067, MO, USA), Beclin-1 (1:1,000; Sigma-Aldrich, cat. no. PRS3613, MO, USA), Atg5 (1:1,000; Sigma-Aldrich, cat. no. A0731, MO, USA), PI3 kinase p85 (1:1,000; Cell Signaling, cat. no. 4257, MA, USA), phospho-PI3 kinase p85/p55 (1:1,000; Cell Signaling, cat. no. 4228, MA, USA), Akt (1:1,000; Cell Signaling, cat. no. 4691, MA, USA), phospho-Akt (Ser473) (1:1,000; Cell Signaling, cat. no. 4060, MA, USA), mTOR (1:1,000; Cell Signaling, cat. no. 2983, MA, USA), phospho-mTOR (1:1,000; Cell Signaling, cat. no. 5536, MA, USA), and β-actin (1:5,000; Sigma-Aldrich, cat. no. A5316, MO, USA). After washing, membranes were incubated with peroxidase-conjugated secondary antibodies (Southern Biotech, Birmingham, AL, United States) for 1 h at room temperature. Finally, the blots were visualized with an enhanced chemiluminescence detection kit (GE Healthcare, Buckinghamshire, United Kingdom) and imaged with a ChemiDoc XRS system (Bio-Rad, Hercules, CA, United States). Quantity One software (Bio-Rad) was used for densitometric analysis.

### Immunofluorescence

The immunofluorescence was performed as previously reported (Huang et al., 2018). Frozen sections of ileal tissue were fixed with 1% paraformaldehyde and then permeabilized in 1% Triton X-100 (Sigma, T9284). The nonspecific binding sites were blocked with 5% normal goat serum for 30 min. Sections were stained with ZO-1 antibody at 1:100 and fluorescence-conjugated secondary antibodies: Alexa Fluor 488-conjugated goat anti-rabbit IgG antibody (Invitrogen) at 1:100 and Alexa Fluor 594-conjugated phalloidin (Invitrogen) at 1:40. The ileal tissues were viewed with a TCS SP5 laser confocal microscope (Leica, Germany) and analyzed using Leica LAS AF 2.3.0 software (Leica Microsystems, Germany).

### Transmission Electron Microscopy

The ileal tissues were harvested and quickly fixed with 2.5% glutaraldehyde in 0.1 mol/L PBS (pH 7.4) for 2 h. Then the samples were fixed with 2% osmium tetroxide, dehydrated in a graded series of alcohol, and finally embedded in Epon 812. An ultramicrotome (Ultract N; Reichert-Nissei, Tokyo, Japan) was used to cut the samples for ultrathin sections. Collected sections were fixed with 2% uranyl acetate and lead citrate and then photographed under a transmission electron microscope (JEM-1400PLUS, Japan).

### Statistical Analysis

SPSS 13.0 software was used for statistical analysis. The data are presented as mean ± SEM. Statistical significance was calculated using one-way analysis of variance (ANOVA) and was set at *p* < 0.05. All reported significance levels represent two-tailed *p*’s.

## Results

### Severe Burn-Induced Intestinal Epithelial ER Stress Promotes the Activation of Autophagy *via* Inhibition of the PI3K/AKT/mTOR Pathway

**Figure 1:**
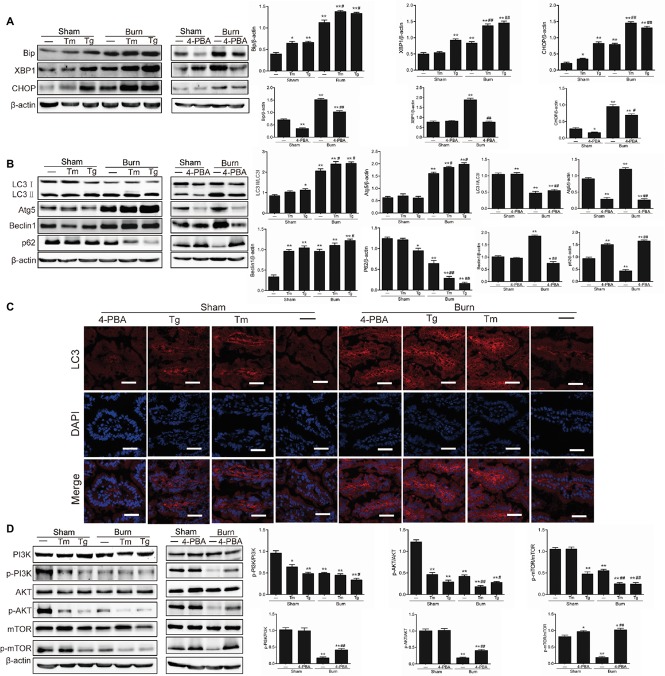
Severe burn-induced endoplasmic reticulum (ER) stress triggers autophagy *via* inhibiting the PI3K/AKT/mTOR pathway. Mice were treated with or without tunicamycin (1.0 mg/kg), thapsigargin (300 ng/kg), and 4-PBA (80 mg/kg), respectively, for 1 h and then inflicted with the sham or 30% TBSA burn. **(A)** The ileal mucosa lysates were taken at 6 h after sham burn or burn injury and then analyzed for the expression of ER stress markers by immunoblot, with β-actin as loading control. Data represent mean ± SEM. ^*^p < 0.05, ^**^p < 0.01, compared with sham-burn group (*n =* 5). ^#^*p* < 0.05, ^##^*p* < 0.01, compared with burn group (*n =* 5). **(B)** The ileal mucosa lysates were analyzed for the expression of autophagy markers by immunoblot. Data represent mean ± SEM. ^*^p < 0.05, ^**^p < 0.01, compared with sham-burn group (*n =* 5). ^#^*p* < 0.05, ^##^*p* < 0.01, compared with burn group (*n =* 5). **(C)** LC3 was stained by immunofluorescence assay. Data are representative of five independent experiments. Scale bar = 20.0 μm. **(D)** The ileal mucosa lysates were analyzed for the expression of PI3K/AKT/mTOR pathway molecules by immunoblot. Data represent mean ± SEM. ^*^p < 0.05, ^**^p < 0.01, compared with sham-burn group (*n =* 5). ^#^*p* < 0.05, ^##^*p* < 0.01, compared with burn group (*n =* 5).

In our previous study, we have found that severe burn injury induced the activation of ER stress in intestinal mucosa, which was accompanied by the activation of autophagy (Huang et al., 2018). However, the exact relationship between ER stress and autophagy in severe burn injury is not clear. To determine the association between ER stress and autophagy in intestinal mucosa, we detected the level of autophagy after the treatment of ER stress inducers (Tm and Tg) or inhibitor (4-PBA) in mice with or without burn injury. We firstly investigated the effect of Tm, Tg, and 4-PBA on ER stress in burned or sham-burned mice. As shown in Figure 1A, the expression levels of the ER stress-related proteins Bip, XBP1, and CHOP were all enhanced after burn injury or Tm or Tg treatment compared with control. Both Tm and Tg further aggravated the severe burn-induced upregulation of Bip, XBP1, and CHOP. However, 4-PBA, a chemical chaperone that was reported to inhibit ER stress in both cell lines and animals (Ozcan et al., 2006; Ozean et al., 2008), decreased the protein levels of Bip, XBP1, and CHOP in severely burned mice, also as illustrated in Figure 1A.

We next determined the effect of ER stress on autophagy in intestinal epithelia. As shown in Figure 1B, the severe burn-induced increase of LC3-II/LC3-I, Atg5, and Beclin 1 were all significantly aggravated by inducing ER stress with Tm and Tg, the ER stress inducers. Conversely, 4-PBA, an ER stress inhibitor, blocked the severe burn-induced increase of LC3-II/LC3-I, Atg5, and Beclin 1. Consistently, the severe burn-induced decrease of p62 was significantly aggravated by Tm or Tg treatment but reversed by inhibiting ER stress with 4-PBA.

We also detected LC3 by an immunofluorescent staining assay in intestinal epithelia. As illustrated in Figure 1C, treatment of severely burned mice with Tm or Tg aggravated the severe burn-induced increase of total LC3 protein. However, the severe burn-induced increase of total LC3 protein was significantly reduced by 4-PBA treatment. Taken together, these data suggest that induction of ER stress promotes the activation of autophagy in the intestinal epithelia of mice suffering from severe burn injury.

Considering the reports that the PI3K/AKT/mTOR signaling pathway plays an important role in the interplay between ER stress and autophagy, we further determined whether the PI3K/AKT/mTOR signaling pathway participates in the autophagy activation by severe burn-induced ER stress in intestinal epithelia. As shown in Figure 1D, the expression of phosphorylated PI3K, AKT, and mTOR was remarkably reduced by the treatment of Tm or Tg to induce ER stress but markedly increased by 4-PBA treatment to inhibit ER stress in mice with severe burn. These results indicate that the suppression of the PI3K/AKT/mTOR signaling pathway is involved in the autophagy activation by severe burn-induced ER stress in intestinal epithelia.

### Activation of Endoplasmic Reticulum Stress is Required for Intestinal Tight Junction Barrier Dysfunction in Severely Burned Mice

**Figure 2:**
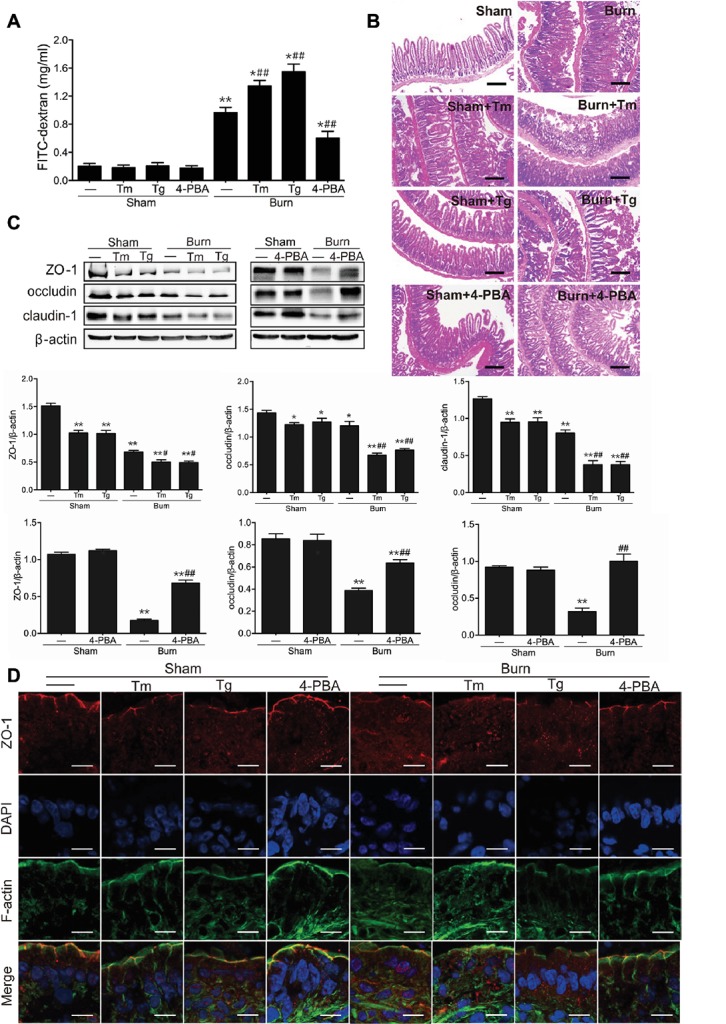
Severe burn-induced intestinal epithelial tight junction (TJ) barrier dysfunction is aggravated by ER stress inducers and alleviated by ER stress inhibitor. Mice were treated as described in Figure 1. **(A)** Intestinal permeability to 4.4 kDa FITC-dextran was measured at 6 h after sham burn or burn injury as described in “Materials and Methods.” Data represent mean ± SEM. ^*^p < 0.05, ^**^p < 0.01, compared with sham-burn group (*n =* 5). ^#^*p* < 0.05, ^##^*p* < 0.01, compared with burn group (*n =* 5). **(B)** Hematoxylin and eosin staining of distal ileal segments was performed at 6 h following sham burn or 30% TBSA burn. Data are representative of five independent experiments. Scale bar = 200 μm. **(C)** Immunoblot was performed to determine the expression of ZO-1, occludin, and claudin-1, with β-actin as loading control. Data represent mean ± SEM. ^*^p < 0.05, ^**^p < 0.01, compared with sham-burn group (*n =* 5). ^#^*p* < 0.05, ^##^*p* < 0.01, compared with burn group (*n =* 5). **(D)** ZO-1 was stained by immunofluorescence assay. Data are representative of five independent experiments. Scale bar = 20.0 μm.

Based on our previous study that severe burn-induced intestinal barrier dysfunction was accompanied by the activation of ER stress (Huang et al., 2018), we sought to determine the contribution of ER stress to the severe burn-induced intestinal epithelial TJ barrier dysfunction. As revealed in Figure 2A, the intestinal paracellular permeability to 4.4 kDa FITC-dextran was significantly increased following severe burn injury. More importantly, the severe burn-induced increase of intestinal paracellular permeability was remarkably aggravated by Tm or Tg treatment but distinctly attenuated by 4-PBA treatment.

To further investigate the role of ER stress activation in the intestinal barrier dysfunction, the damage of the intestinal mucosa was examined by hematoxylin and eosin staining. As shown in Figure 2B, in control mice, the intestinal epithelia were intact, and the villi were in good order. However, in the intestinal mucosa of burned mice, the epithelial cells were exfoliated and accompanied by exposed lamina propria, necrosis, and inflammatory cell infiltration. Notably, the severe burn-induced histological damages were aggravated by Tm or Tg treatment and dramatically alleviated by 4-PBA.

Considering the critical roles of TJ proteins in the regulation of intestinal epithelial barrier function, we then investigated whether inducing or inhibiting ER stress affected TJ protein expression and ZO-1 localization in severely burned mice. As shown in Figure 2C, Tm or Tg treatment caused a remarkable decline in the expression of ZO-1 and claudin-1 in mice with or without burn injury. The protein level of occludin was significantly reduced by Tm or Tg treatment in severely burned mice. However, 4-PBA treatment reversed the severe burn-induced decrease of ZO-1, occludin, and claudin-1.

ZO-1 is indispensable in the maintenance of the intestinal leaky pathway and the regulation of paracellular permeability of the TJ, and its defects directly result in the failure of TJ formation (Umeda et al., 2006). Our previous studies have demonstrated that ZO-1, as an important TJ protein in the maintenance and regulation of the intestinal leaky pathway, has been relocalized after severe burn injury (Chen et al., 2012; Huang et al., 2018). Thus, we further sought to assess the influence of ER stress on morphological changes of the ZO-1 protein in severely burned mice by immunofluorescent assay. As illustrated in Figure 2D, ZO-1 (shown as red) was localized to the epithelial TJs and can be seen as a series of ordered bright red spots at the apical junctions in the control group. Treatment of mice with Tm or Tg resulted in ZO-1 relocalization and worsened the severe burn-induced reorganization of ZO-1, which can be seen as a loss of bright red spots at the apical junctions. However, treatment of mice with 4-PBA did not obviously affect the morphological distribution of ZO-1 in sham-burned mice, but evidently alleviated the relocalization of ZO-1 in severely burned mice.

### Autophagy Activation Is Involved in Intestinal Tight Junction Barrier Dysfunction Induced by Severe Burn Injury

**Figure 3:**
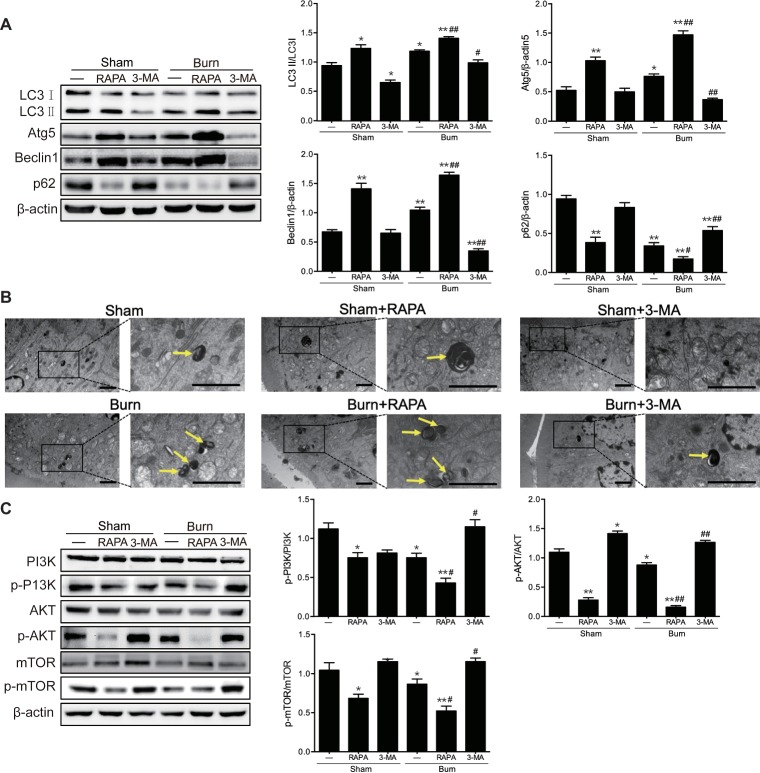
Severe burn-induced autophagy activation is exacerbated by autophagy inducer and suppressed by autophagy inhibitor. Mice were treated with or without rapamycin (4 mg/kg) and 3-methyladenine (15 mg/kg), respectively, for 1 h and then given the sham or 30% TBSA burn. **(A)** The ileal mucosa lysates were taken at 6 h after sham burn or burn injury and then analyzed for the expression of autophagy markers by immunoblot, with β-actin as loading control. Data represent mean ± SEM. ^*^p < 0.05, ^**^p < 0.01, compared with sham-burn group (*n =* 5). ^#^*p* < 0.05, ^##^*p* < 0.01, compared with burn group (*n =* 5). **(B)** Representative autophagosome (yellow arrow) images of intestinal epithelial cells from sham-burned or burned mice under transmission electron microscopy (TEM). Scale bar = 2.0 μm. *n =* 5. **(C)** The ileal mucosa lysates were analyzed for the expression of PI3K/AKT/mTOR pathway molecules by immunoblot. Data represent mean ± SEM. ^*^p < 0.05, ^**^p < 0.01, compared with sham-burn group (*n =* 5). ^#^*p* < 0.05, ^##^*p* < 0.01, compared with burn group (*n =* 5).

In our previous study, we have found that the severe burn-induced TJ barrier dysfunction was accompanied by the autophagy activation in the ileum of burned mice (Huang et al., 2018). Thus, we sought to determine whether the activation of autophagy was involved in the intestinal TJ barrier dysfunction caused by severe burn injury. First, we treated burned or sham-burned mice with rapamycin, an mTOR inhibitor and classical autophagy inducer, or 3-MA, an autophagy inhibitor suppressing the activity of class III PI3K, to trigger or suppress autophagy activation, respectively. As shown in Figure 3A, treatment of burned or sham-burned mice with rapamycin resulted in a striking increase in LC3П/LC3І ratio and the expression of both Atg5 and Beclin 1, and a decrease in p62 expression in the intestinal epithelia. On the contrary, 3-MA treatment caused a marked decrease in LC3П/LC3І ratio and the expression of both Atg5 and Beclin 1, and elevation of p62 expression in the intestinal epithelia of burned mice. Consistent with these results, rapamycin treatment significantly increased the number of autolysosome and autophagosomes in the intestinal epithelial cells of burned or sham-burned mice, whereas 3-MA treatment remarkably decreased the number of autolysosome and autophagosomes in mice inflicted with severe burn injury, as illustrated in Figure 3B. In addition, as shown in Figure 3C, the protein expression of phosphorylated PI3K, phosphorylated AKT, and phosphorylated mTOR were all remarkably reduced by the treatment of rapamycin but markedly increased by 3-MA.

**Figure 4:**
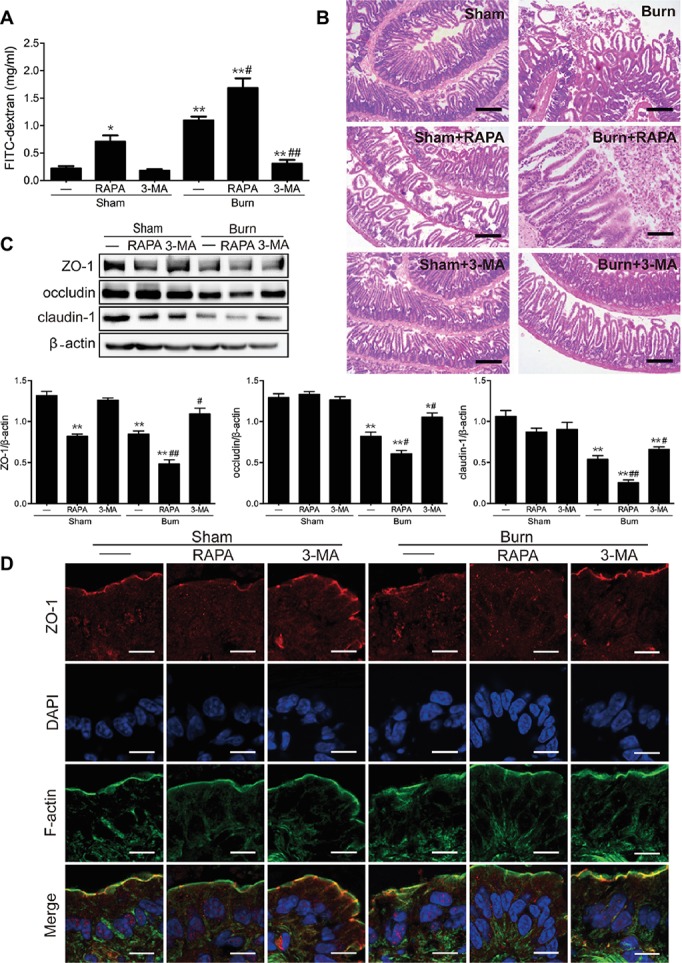
Severe burn-induced intestinal epithelial TJ barrier dysfunction is exacerbated by rapamycin and ameliorated by 3-MA. Mice were treated as described in Figure 3. **(A)** Intestinal permeability to 4.4 kDa FITC-dextran was measured at 6 h after sham burn or burn injury as described in “Materials and Methods.” Data represent mean ± SEM. ^*^p < 0.05, ^**^p < 0.01, compared with sham-burn group (*n =* 5). ^#^*p* < 0.05, ^##^*p* < 0.01, compared with burn group (*n =* 5). **(B)** Hematoxylin and eosin staining of distal ileal segments was performed at 6 h following sham burn or 30% TBSA burn. Data are representative of five independent experiments. Scale bar = 200 μm. **(C)** Immunoblot was performed to determine the expression of ZO-1, occludin, and claudin-1, with β-actin as loading control. Data represent mean ± SEM. ^*^p < 0.05, ^**^p < 0.01, compared with sham-burn group (*n =* 5). ^#^*p* < 0.05, ^##^*p* < 0.01, compared with burn group (*n =* 5). **(D)** ZO-1 was stained by immunofluorescence assay. Data are representative of five independent experiments. Scale bar = 20.0 μm.

We then evaluated the effect of autophagy activation on epithelial TJ barrier function in the ileum of mice with or without severe burn. As revealed in Figure 4A, inducing autophagy with rapamycin significantly elevated paracellular permeability to FITC-dextran in sham-burned mice and distinctly exacerbated the severe burn-induced increase of paracellular permeability in burned mice. In contrast, inhibiting autophagy with 3-MA had no significant effect on paracellular permeability but markedly dampened severe burn-induced increase of paracellular permeability in the ileum of mice suffering from severe burn injury. Moreover, we further determined the influence of autophagy activation on severe burn-induced histological damage of intestinal mucosa. As illustrated in Figure 4B, the damage of ileal mucosa was obviously in burned mice when compared to the sham-burned mice. Notably, rapamycin treatment remarkably exasperated the burn-induced damage of ileal mucosa. In contrast, treatment of mice with 3-MA did not affect the histological morphology of ileal mucosa in sham-burned mice, but evidently alleviated the severe burn-induced histological damage of ileal mucosa. These results imply that activation of autophagy aggravates the intestinal epithelial barrier dysfunction induced by severe burn, whereas inhibition of autophagy attenuates the severe burn-induced intestinal epithelial barrier dysfunction.

Having confirmed that the autophagy activation is involved in severe burn-induced intestinal barrier dysfunction, we next investigated the effect of autophagy activation on the expression of TJ proteins in the ileal mucosa of mice inflicted with or without burn injury. As shown in Figure 4C, rapamycin treatment significantly reduced the expression of ZO-1 and claudin-1 in sham-burned mice and distinctly exacerbated the severe burn-induced decrease of ZO-1, occludin, and claudin-1 expression in burned mice. On the contrary, 3-MA treatment did not remarkably alter the expression of ZO-1, occludin, and claudin-1 in sham-burned mice, but almost completely blocked the severe burn-induced decrease of ZO-1, occludin, and claudin-1 expression in burned mice. Moreover, the effect of autophagy activation on the distribution and localization of ZO-1 in the ileum was examined by immunofluorescent assay coupled with confocal microscopy. As demonstrated in Figure 4D, ZO-1 exhibited a series of red spots that were bright and ordered at the apical junctions in sham-burned mice. In contrast, the burned mice showed disorganized and random ZO-1 staining. Treatment of mice with rapamycin caused relocalization of ZO-1 in sham-burned mice and worsened the severe burn-induced ZO-1 reorganization, which can be seen in an obvious thinning and discontinuity of staining. However, 3-MA treatment did not obviously affect the morphological distribution of ZO-1 in sham-burned mice, but evidently alleviated the severe burn-induced relocalization of ZO-1.

## Discussion

The intestinal epithelial barrier plays an essential role in maintaining intestinal homeostasis. Burn injury, as one of the most common traumas, results in a mesenteric vasoconstriction and a lack of oxygen to the gut. Severe burn-induced damage participates in the occurrence and progress of gut barrier dysfunction and can activate the host’s innate immune response. Thus, it results in the failure of the mucosa to act as an efficient barrier against luminal toxins, pathogenic organisms, and antigenic molecules (Saadia et al., 1990). Our previous studies revealed that severe burn increased the intestinal paracellular permeability to 4.4 kDa FITC-dextran, caused histological damage of intestinal mucosa, and altered TJ protein expression and ZO-1 localization in mice (Chen et al., 2012; Huang et al., 2018), indicating that severe burn can induce the intestinal TJ barrier disruption. However, the underlying molecular mechanisms have not been fully elucidated so far.

Severe burn injury of the skin can induce intestinal dysfunction and pathology, including stress ulcers and increased intestinal permeability (Deitch, 1990; Epstein et al., 1991). Previous studies have reported that severe burn injury results in a significant reduction in blood flow to the small intestine (Kregel et al., 1988), which further leads to intestinal mucosal barrier dysfunction and induced ischemia at the villus tip (Simon, 1994; Hall et al., 2001; Yu et al., 2010). Intestinal ischemia causes the damage of intestinal mucosa (Moriwaki et al., 1993).

The process of protein folding is especially sensitive to endogenous or exogenous stress. The accumulation of unfolded proteins in the ER causes ER stress and induces the UPR, which alleviates stress through upregulating protein folding and degradation pathways in the ER inhibiting protein synthesis (Rutkowski and Kaufman, 2004; Schroder and Kaufman, 2005). Severe burn-induced intestinal ischemia leads to oxidative stress and insufficient exogenous blood supply, resulting in limited nutrient delivery, and could induce further ER stress. Severe burn has been documented to induce ER stress in some visceral organs, suggesting that ER stress is an important cellular response to burn injury and is associated with organ damage and dysfunction (Gauglitz et al., 2010; Jeschke et al., 2012; Song et al., 2014). ER stress was reported to be associated with intestinal epithelial dysfunction in inflammatory bowel disease (Kaser et al., 2010; Adolph et al., 2013; Cao, 2015; Luo and Cao, 2015; Chotikatum et al., 2018). Several clinical studies have also shown evidence for enhanced ER stress in the intestines of patients with inflammatory bowel disease (Hu et al., 2007; Shkoda et al., 2007; Kaser et al., 2008). Although the UPR is initially to compensate for damage, chronic ER stress and defective UPR impair intestinal homeostasis, which may finally induce intestinal inflammation (Cao, 2016).

The epithelial TJs that control paracellular permeability are complex structures composed of the TJ proteins, such as transmembrane proteins (e.g., occludin and claudins), and peripheral membrane proteins (e.g., ZO-1). The localization and interactions of TJ proteins are critical in maintaining intestinal barrier function. Thus, alterations in TJ proteins are associated with changes in intestinal permeability. A study reported that acrolein-induced intestinal epithelium dysfunction is associated with a decrease/redistribution of TJ proteins and intestinal ER stress (Chen et al., 2017). We previously observed that intestinal barrier dysfunction was associated with intestinal ER stress in response to severe burn (Huang et al., 2018). However, the exact role of ER stress in the burn-induced intestinal barrier dysfunction needed to be determined. Thus, in this study, we further demonstrate that activating ER stress with specific inducers Tm or Tg significantly aggravates the severe burn-induced intestinal epithelial TJ barrier dysfunction, whereas suppressing ER stress with specific inhibitor 4-PBA markedly alleviates the intestinal epithelial TJ barrier dysfunction caused by severe burn. Therefore, it is strongly suggested that the burn-triggered ER stress is an important cellular mechanism involved in the intestinal epithelial TJ barrier dysfunction induced by severe burn injury.

Autophagy is stimulated during situations of hypoxia, viral infection, nutrient deprivation, and ER stress (Avivar-Valderas et al., 2011; Hart et al., 2012). Accumulating evidence shows that ER stress induces autophagy under the condition of excessive accumulation of unfolded or misfolded proteins. Our previous study has demonstrated that ER stress and autophagy are both associated with the intestinal barrier dysfunction caused by severe burn injury (Huang et al., 2018). However, the interplay between ER stress and the activation of autophagy in intestinal epithelial cells following severe burn injury remains unknown. Here in the present study, we show that severe burn injury-induced ER stress results in the activation of autophagy *via* downregulation of the PI3K/AKT/mTOR signaling pathway in intestinal epithelial cells. This is consistent with the other studies revealing that activation of PI3K/AKT/mTOR results in excessive autophagy (Aki et al., 2003; Qin et al., 2014).

A basal level of autophagy is considered cytoprotective when compared with apoptosis (Boya et al., 2005). However, a high level of autophagy is induced under stress conditions, which can indeed facilitate cell death in specific contexts (Denton et al., 2015). Thus, the biological significance of autophagy is still controversial. Of course, the regulatory role of autophagy in epithelial barrier dysfunction also remains to be clarified. Recent studies have shown that autophagy is a cytoprotective response to damage of the intestinal tract (Zhang et al., 2017a,b). Similarly, autophagy induced by nutrient starvation has been reported to regulate the conductivity of small uncharged molecules and cations to enhance intestinal barrier dysfunction *via* targeting claudin-2 protein degradation in Caco-2 cells (Nighot et al., 2015). However, a recent study has reported that autophagy activation results in endothelial barrier dysfunction, and inhibiting autophagy protects against endothelial barrier dysfunction by suppressing cadherin disassembly and actin stress fiber formation (Slavin et al., 2018). In this study, we demonstrate that inducing autophagy with rapamycin leads to the intestinal barrier dysfunction in sham-burned mice and aggravates the intestinal barrier dysfunction in severely burned mice, whereas inhibiting autophagy with 3-MA attenuates the severe burn-induced intestinal barrier dysfunction. Thus, it is suggested that autophagy activation contributes to the intestinal barrier dysfunction induced by severe burn injury.

It has been well documented that TJ proteins are crucial to the maintenance of intestinal epithelial barrier function (Turner, 2009). In this study, we reveal that inducing autophagy by rapamycin distinctly exacerbates the burn-induced decrease of TJ proteins ZO-1, occludin, and claudin-1, and worsens the burn-induced ZO-1 relocalization. In contrast, inhibiting autophagy by 3-MA dampens the burn-induced reduction of ZO-1, occludin, and claudin-1, and alleviates the burn-induced relocalization of ZO-1. In accordance with our present findings, the latest studies have demonstrated that autophagy activation results in the reduction and redistribution of TJ proteins ZO-1, occludin, and claudin-5 in endothelial cells, leading to the endothelial barrier disruption (Chan et al., 2018; Zhang et al., 2018). Thus, when all this is taken together, it is implied that autophagy activation is involved in the burn-induced intestinal TJ barrier dysfunction, at least in part, by inducing both reduction and reorganization of TJ proteins.

In summary, it is concluded that severe burn injury triggers ER stress in intestinal epithelial cells, which in turn, induces autophagy activation *via* inhibiting the PI3K/AKT/mTOR signaling pathway. The ER stress-induced activation of autophagy contributes to the severe burn-induced intestinal epithelial TJ barrier dysfunction by causing both reduction and reorganization of TJ proteins. These novel findings, for the first time, suggest that the ER stress-autophagy axis is involved in the intestinal epithelial TJ barrier dysfunction induced by severe burn injury. Therefore, inhibition of either ER stress or autophagy may be helpful to restore the intestinal epithelial TJ barrier following severe burn injury.

## Data Availability

The raw data supporting the conclusions of this manuscript will be made available by the authors, without undue reservation, to any qualified researcher.

## Ethics Statement

The animal studies were approved by the Animal Care and Use Committee of the Third Military Medical University (Army Medical University), Chongqing, China. And all animal procedures were performed in adherence to protocols approved by the Ethics Committee of Southwest Hospital, Third Military Medical University (Army Medical University).

## Author Contributions

YH performed the experiments, drafted the manuscript, and prepared the figures. YW and YF drafted parts of the manuscript and prepared the figures. PW and XH performed parts of the experiments. HR and FW designed the experiments and revised the manuscript.

### Conflict of Interest Statement

The authors declare that the research was conducted in the absence of any commercial or financial relationships that could be construed as a potential conflict of interest.
